# Sustained Low Serum Substance P Levels in Non-Surviving Septic Patients

**DOI:** 10.3390/ijms18071531

**Published:** 2017-07-15

**Authors:** Leonardo Lorente, María M. Martín, Antonia Pérez-Cejas, José Ferreres, Jordi Solé-Violán, Lorenzo Labarta, César Díaz, Alejandro Jiménez

**Affiliations:** 1 Intensive Care Unit, Hospital Universitario de Canarias, Ofra, s/n. La Laguna, Tenerife 38320, Spain; 2 Intensive Care Unit, Hospital Universitario Nuestra Señora Candelaria, Crta Rosario s/n. Santa Cruz Tenerife 38010, Spain; mar.martinvelasco@gmail.com; 3 Laboratory Deparment, Hospital Universitario de Canarias, Ofra, s/n. La Laguna, Tenerife 38320, Spain; aperezcejas@gmail.com; 4 Intensive Care Unit, Hospital Clínico Universitario de Valencia, Avda, Blasco Ibáñez n° 17-19, Valencia 46004, Spain; ferreresj@gmail.com; 5 Intensive Care Unit, Hospital Universitario Dr. Negrín, Barranco de la Ballena s/n. Las Palmas de Gran Canaria 35010, Spain; jsolvio@gobiernodecanarias.org; 6 Intensive Care Unit, Hospital San Jorge de Huesca, Avenida Martínez de Velasco n° 36, Huesca 22004, Spain; llabarta@salud.aragon.es; 7 Intensive Care Unit, Hospital Insular, Plaza Dr. Pasteur s/n. Las Palmas de Gran Canaria 35016, Spain; incaicos@yahoo.es; 8 Research Unit, Hospital Universitario de Canarias, Ofra, s/n. La Laguna, Tenerife 38320, Spain; ajimenezsosa@gmail.com

**Keywords:** substance P, patients, sepsis, mortality, outcome

## Abstract

Previously, researchers found higher serum substance P (SP) concentrations in survivors of severe sepsis than in non-survivors at the time of severe sepsis diagnosis. The objectives of our current study were to determine whether there is an association between serum SP levels during the first week and sepsis mortality, sepsis severity, serum levels of tumor necrosis factor (TNF)-α and interleukin (IL)-10, and whether serum SP levels during the first week could be used as a biomarker of sepsis mortality. We determined serum concentration of SP, TNF-α, and IL-10 at days 1, 4, and 8. The end-point of the study was mortality at 30 days. We found that **n**on-survivor (*n* = 104) compared to survivor patients (*n* = 206) showed lower serum SP levels at days 1, 4, and 8 (*p <* 0.001). Multiple logistic regression analyses showed an association between 30-day mortality and serum SP levels at days 1, 4, and 8 (*p* < 0.001) controlling for SOFA score, diabetes mellitus, age, and lactic acid levels. The most interesting findings of our study were that there is an association between serum SP levels during the first week and sepsis mortality, and that serum SP levels during the first week could be used as a biomarker of sepsis mortality.

## 1. Introduction

Sepsis carries a large number of deaths and health care costs annually [1,2]. Tachykinin family includes substance P (SP), neurokinin A (NKA), neurokinin B (NKB), and endokinins [3,4,5,6,7,8,9,10,11,12]. Tachykinins are present in the peripheral and central nervous systems, respiratory system, urinary system, immune system, gut, and blood vessels. Tachykinins are involved in different biological processes, such as transmission of nociceptive responses, airway contraction, salivary secretion, smooth muscle contraction, inflammation, vasodilatation, and plasma protein extravasation. SP is involved in different diseases, such as asthma, psoriasis, inflammatory bowel disease, anxiety, migraine, psychosis, and central and peripheral nervous systems injury [3,4,5,6,7,8,9,10,11,12]. 

SP is a polypeptide that is encoded by TAC1 gene, and its effects are mediated binding to NK_1_ receptor (NK_1_R) (which is widely expressed in many tissues and cells) [3,4,5,6,7,8,9,10,11,12]. SP is considered one of the major initiators of neurogenic inflammation.

The role of SP in sepsis remains unclear. On the one hand, in some studies it was found that SP could play a role in the inflammatory response to sepsis by the release of pro-inflammatory cytokines as interleukin (IL)-1, IL-6, and tumor necrosis factor (TNF)-α [13,14,15,16]. On the other hand, in other studies it was found that SP could have anti-inflammatory effects by reducing TNF-α, IL-6, and inducible nitric oxide synthase (iNOS), and increasing IL-10 [17,18]. Besides, according to the findings of another studies, SP could play a role in the microorganism clearance modulating the phagocytosis capacity [19,20,21,22].

Circulating SP concentrations in septic patients have not been well studied [23,24,25,26]. In one study with 61 septic patients, authors found higher serum SP concentrations in septic patients compared to healthy controls, and in non-survivor compared to survivor patients during the final phase of sepsis [23]. In another study with 42 septic patients were found lower plasma SP concentrations in septic patients compared to healthy controls [24]. Other authors have shown an 80% reduction in SP expression in myenteric plexus neurons of small bowel from patients with peritonitis compared to patients without peritonitis [25]. Our team has determined serum SP levels at the time of severe sepsis diagnosis in 238 severe septic patients [26], and we found higher serum SP concentrations in survivor than in non-survivor severe septic patients. We also found that serum SP concentrations at baseline were associated with 30-day mortality. The objectives of our current research (increasing the sample size to 310 severe septic patients, and determining serum SP levels at days 1, 4, and 8 of severe sepsis diagnosis) were to determine whether there is an association between serum SP levels during the first week and sepsis mortality, sepsis severity, serum levels of TNF-α and IL-10 (as pro-inflammatory and anti-inflammatory cytokine, respectively), and whether serum SP levels during the first week could be used as a biomarker of sepsis mortality. The interest of our current study lies in that if these associations are find, then serum SP levels determination could be used in clinical practice to estimate the prognosis of these patients, and one new approach in the research for the treatment of these patients could be suggested.

## 2. Results

Comparisons on demographic and clinical characteristics between non-survivor (*n* = 104) and survivor severe septic patients (*n* = 206) at moment of severe sepsis diagnosis are showed in Table 1. We did not find statistically significant differences between non-survivor and survivor severe septic patients on sex, COPD, chronic renal failure, ischemic heart disease, PaO_2_/FIO_2_ ratio, leukocytes, bilirubin, bloodstream infection, site of infection, microorganism responsible of the infection, and serum levels of TNF-α. However, we found that non-survivors with severe sepsis compared to survivors had lower platelet count, and higher age, SOFA score, lactic acid, INR, creatinine, aPTT, APACHE-II score, serum IL-10 levels, and rate of diabetes mellitus. In addition, non-survivors had lower serum levels of SP at the time of severe sepsis diagnosis (*p <* 0.001).

**Figure 1:**
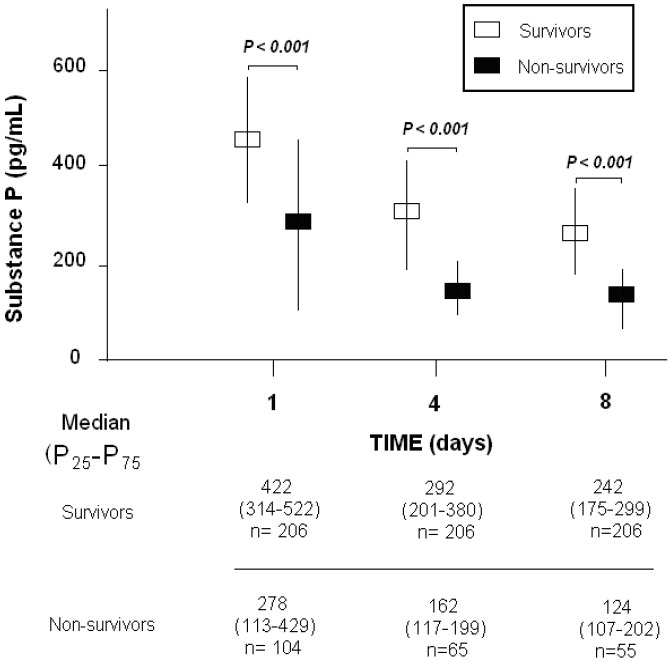
Serum substance P levels in survivor and non-survivor severe septic during the first week.

Table 2 compares changes in serum levels of SP, TNF-α and IL-10, and SOFA score during the first week of severe sepsis. Non-survivors in respect to survivors showed lower serum levels of SP (Figure 1), and higher SOFA score and serum levels of IL-10 during the first week. In addition, we found higher serum levels of TNF-α in non-survivor as compared to survivor septic patients at days 4 and 8 of severe sepsis diagnosis.

Table 3 shows the correlations between serum levels of SP, TNF-alpha and IL-10, and SOFA score during the first week of severe sepsis. There were found a statistically significant negative correlation between serum SP levels and SOFA score at day 4 of severe sepsis diagnosis, and a trend of not statistically significant at days 1 and 8.

**Figure 2:**
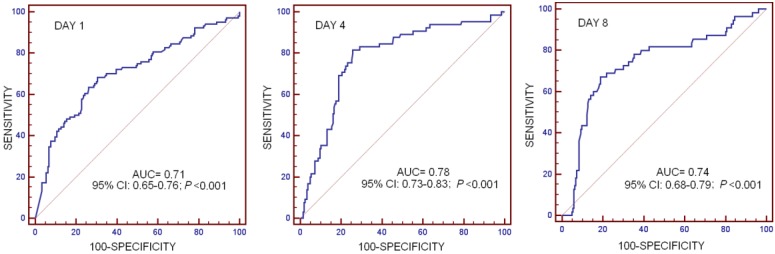
Curves of receiver operation characteristic analysis using serum Substance P levels at days 1, 4, and 8 as a predictor of mortality at 30 days in severe septic patients.

In Table 4 and Figure 2 appears ROC curve analyses for serum SP levels at days 1, 4, and 8 to predict 30-day mortality. We found that serum SP levels at day 1 (*p* < 0.001), day 4 (*p* < 0.001), and day 8 (*p* < 0.001) could predict 30-day mortality.

Table 5 shows multiple logistic regression analyses, and we found an association between 30-day mortality and serum SP levels at days 1, 4, and 8 (*p* < 0.001) controlling for SOFA score, diabetes mellitus, age, and lactic acid levels. 

**Figure 3:**
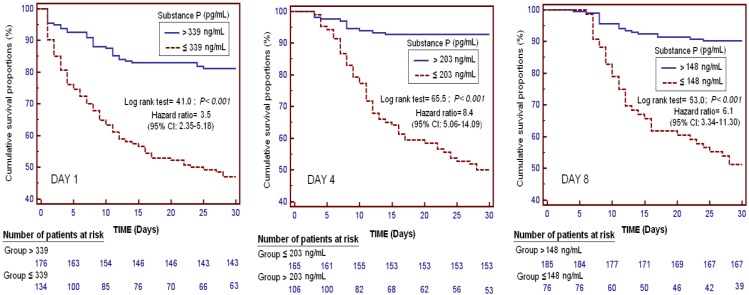
Survival curves at 30 days using serum substance P levels higher or lower than 339 pg/mL.

In Figure 3 appears Kaplan–Meier survival analysis at days 1, 4, and 8 of severe sepsis diagnosis according to different serum SP levels. We found a higher risk of death risk in patients with serum SP levels < 339 pg/mL at the time of severe sepsis diagnosis than in patients with higher concentrations (Hazard Ratio = 3.5; 95% CI = 2.35–5.18; *p <* 0.001), in patients with serum SP levels < 203 pg/mL at day 4, and in patients with serum SP levels < 148 pg/mL at day 8. 

## 3. Discussion

To our knowledge, the current study is the largest reporting data on circulating SP levels from septic patients. The most interesting findings of our study were that there is an association between serum SP levels during the first week and sepsis mortality, and that serum SP levels during the first week could be used as a biomarker of sepsis mortality.

In a previous study [26], we found higher serum SP levels at moment of severe sepsis diagnosis in survivor than in non-survivor severe septic patients; thus, a novel finding of our current study was that survivor severe septic patients showed persistently higher serum SP levels during the first week than non-survivors. We have not determined serum SP levels in healthy controls; however, the serum SP levels found in our survivor and non-survivor severe septic patients were lower than those described from healthy volunteers in the data set of kit used for the assay (mean 628, and range 402–1576 pg/mL). Our findings are in accordance with those from the study by Arnalic et al. with 42 septic patients, in which were found lower plasma SP concentrations in septic patients compared to healthy controls [24]; and with those from the study by Jacob et al., in which was found lower SP expression in small bowel from patients with than without peritonitis [25]. On the other hand, our findings contradict with those of the study by Beer et al. with 61 septic patients, in which were found higher serum SP concentrations in septic patients compared to healthy controls and in non-survivor compared to survivor patients during the final phase of sepsis [23].

In our previous study, an association was found between sepsis mortality and serum SP levels at moment of severe sepsis diagnosis [26]; then, the association between sepsis mortality and serum SP levels during the first week found in our current study is another new point. In addition, the capacity of serum SP levels at moment of severe sepsis diagnosis to predict 30-day mortality was found in our previous study [26]; then, another novel finding of our current study was that serum SP levels at days 4 and 8 also could predict 30-day mortality.

The role of SP in sepsis remains unclear. On the one hand, in some studies was found that SP could play a role in the inflammatory response to sepsis by the release of pro-inflammatory cytokines as IL-1, IL-6, and TNF-α [13,14,15,16]. On the other hand, in other studies it has been found that the intravenous administration of SP in animal models with spinal cord injury decreased TNF-alpha, IL-6, and iNOS, and increasied IL-10 [17,18]. However, in our study we found no correlation between serum levels of SP, TNF-α, and IL-10. Besides, SP could pay a role in the phagocytosis; and therefore, in the microorganism clearance and infection control [19,20,21,22]. In a study by Verdrengh et al., *Staphylococcus aureus* was administered intravenously to neurokinin-1 receptor (NK-1R) knockout mice (without receptors to substance P) and control mice, and found that NK-1R knockout mice compared to control mice showed fewer phagocytose bacteria capacity of macrophages, higher burden of staphylococci in the kidneys, more severe arthritic lesions with more severe synovitis and cartilage/bone destruction, and higher 11-day post-infection mortality rate [19]. In a study by Yang et al. it was found that the administration of NK-1R antagonist reduces pulmonar bacterial clearance and survival in mice undergoing to traumatic brain injury and intrapulmonary infection with *Pseudomonas aeruginosa* [20]. In a study by Kincy et al., Salmonella was administered orally to mice previously or not treated with an NK-1R antagonist and there was found that mice pretreated with the NK-1R antagonist compared to mice control developed more severe salmonellosis and had lower survival rates [21]. In a study by Lighvani et al., it was found that mice pretrated with a NK-1R antagonist before a corneal infection with *Pseudomonas aeruginosa* showed more severe disease with higher corneal perforation rate that control mice [22]. 

We think that the finding of our study about that non-survivor severe septic patients showed persistently lower serum SP levels during the first week than survivors could represents that non-survivor patients showed a lower phagocytosis capacity and microorganism clearance. Besides, according to the findings of those studies in animal models with SP administration, it is possible that non-survivor patients showed a lower inflammatory state. More research could be interesting to confirm our findings due to that in that case, serum SP levels determination could be used in clinical practice to estimate the prognosis of these patients, and one new approach in the research for the treatment of these patients could be suggested.

Our study had some limitations. First, we have not determined serum SP levels in healthy volunteers, septic patients without criteria of severe sepsis, and other critically ill patients; however, the objective of our study was not to determine whether severe sepsis influenced serum SP levels, but rather was to determine whether there is an association between serum SP levels during the first week and sepsis mortality, and whether serum SP levels during the first week could be used as a biomarker of sepsis mortality. In addition, serum SP levels found in our survivor and non-survivor severe septic patients were lower than those described from healthy volunteers in the data set of kit used for the assay (and were lower in our non-survivor than in survivor septic patients). Second, the determination of serum substance P levels during more days of follow-up could be interesting. Third, we have not evaluated the phagocytosis capacity in survivor and non-survivor septic patients. Fourth, we have not determined circulating levels of interleukin-6 and c-reactive protein. Fifth, we did not calculate sample size in our study although was enough to find that there is an association between serum SP levels during the first week and sepsis mortality, and that serum SP levels during the first week could be used as biomarker of sepsis mortality. Sixth, there is a new sepsis definition [27]; however, we used the same sepsis definition in both works of our team [28].

## 4. Methods

### 4.1. Design and Subjects

A prospective, multicenter, observational study was carried out with 321 severe septic patients in Intensive Care Units from six Spanish hospitals: Clínico Universitario de Valencia (Valencia), Insular (Las Palmas de Gran Canaria), San Jorge (Huesca), Universitario de Canarias (La Laguna. Tenerife), Universitario Dr. Negrín (Las Palmas de Gran Canaria), and Universitario Nuestra Señora de Candelaria (Santa Cruz de Tenerife). The study was approved by the Institutional Ethic Review Boards of the six hospitals. Patients or family members signed the informed consent to participate in the study. The study adheres to the World Medical Association Declaration of Helsinki regarding ethical conduct of research involving human subjects.

The criterion for inclusion was severe sepsis according to the International Sepsis Definitions Conference [28]. Criteria for exclusion were: pregnancy, lactation, age < 18 years, steroid, immunosuppressive or radiation therapy, human immunodeficiency virus (HIV), white blood cell count <1000/µL, solid or hematological tumor. 

Previously, we determined other circulating biomarkers in some of those patients [29,30,31,32]. In a previous work, we analyzed serum SP levels in 238 severe septic patients at the time of severe sepsis diagnosis (within 2 h of the diagnosis of severe sepsis) [26]. In the current work, we have analyzed serum SP levels in 310 severe septic patients during the first week of severe sepsis. 

The end-point of the study was 30-day mortality. We also collected the following variables: sex, age, ischemic heart disease, diabetes mellitus, chronic obstructive pulmonary disease (COPD), chronic renal failure (defined as glomerular filtration rate lower than 60 mL/min per 1.73 m^2^), Sepsis-related Organ Failure Assessment [SOFA] score [33], platelets, pressure of arterial oxygen/fraction inspired of oxygen (PaO_2_/FIO_2_), leukocytes, lactic acid, international normalized ratio (INR), creatinine, bilirubin, activated partial thromboplastin time (aPTT), Acute Physiology and Chronic Health Evaluation II (APACHE II) score [34], bloodstream infection, empiric antimicrobial treatment, microorganism responsible, and site of infection.

### 4.2. Determination of Serum Concentrations of SP, Tumor Necrosis Factor (TNF)-α, and Interleukin (IL)-10

Blood samples were collected from patients on day 1, 4, and 8 of severe sepsis diagnosis to determine serum concentrations of SP, TNF-alpha, and IL-10. Blood samples were deposited in serum separator tubes, then blood samples were allowed to clot for 10 min at room temperature, later were centrifuged at 1000× *g* for 15 min and the supernatant was immediately stored in aliquot at −80 °C until assayed to the end of the recruitment process. Samples were transported between different locations in refrigerated boxes with dry ice. The determinations of serum concentrations were performed at the end of the recruitment process in the Laboratory Department of the Hospital Universitario de Canarias (La Laguna, Tenerife, Spain) blinded to clinical data. 

Serum SP levels were assayed by specific Enzyme Linked Immunosorbent Assay (ELISA) according to the manufacturer’s instructions (R&D Systems, Abingdon, UK). Serum concentrations of TNF-alpha and IL-10 were determined using solid-phase chemiluminescent immunometric assays using Immulite^®^ (Siemens Healthcare Diagnostics Products, Llanberis, UK). The intra-assay coefficient of variation (CV) were 9%, 3.6%, and 9.9%, respectively. The inter-assay CV were 15%, 6.5%, and 9.9%, respectively. The detection limits for the assays were 25, 1.7, and 1.0 pg/mL, respectively. 

### 4.3. Statistical Methods

We reported categorical variables as frequencies (and percentages), and continuous variables as medians (and interquartile ranges); and we used chi-square test and Mann–Whitney U test, respectively, for the comparison between patient groups. 

We did receiver operating characteristic (ROC) analyses using serum SP levels at days 1, 4, and 8 as prognostic variables; and survival at 30 days as classification variable. Youden J index was used to select the optimal prognostic cut-off value of serum SP levels for days 1, 4, and 8. We made multiple logistic regression analyses to test the association between 30-day mortality and serum SP levels at days 1, 4, and 8, controlling for SOFA score, lactic acid levels, diabetes mellitus, and age. To measure the clinical impact of prognostic variables were calculated hazard ratio and its 95% confidence intervals (CI). 

We analyzed 30-day survival using Kaplan–Meier method; and we used serum SP levels higher or lower than 339 pg/mL at day 1, 203 pg/mL at day 4, and 148 pg/mL at day 8 as the independent variables, survival at 30 days as the dependent variable, and log-rank test for the comparison. We used those serum SP levels values (339, 203, and 148 pg/mL) because are the optimal prognostic cut-off values according to Youden J index calculations.

Spearman’s rank coefficient was used to determine the correlation between serum levels of SP, TNF-α and IL-10, and SOFA score at days 1, 4, and 8. Values of *p* less than 0.05 were considered statistically significant; besides, Bonferroni correction was used in multiple comparisons. We performed statistical analyses by SPSS 17.0 (SPSS Inc., Chicago, IL, USA) and NCSS 2000 (Kaysville, UT, USA).

## 5. Conclusions

The current study is the largest reporting data on circulating SP levels from septic patients. The most interesting findings of our study were that there is an association between serum SP levels during the first week and sepsis mortality, and that serum SP levels during the first week could be used as biomarker of sepsis mortality based on findings in our population.

## Figures and Tables

**Table 1:** Comparisons between non-survivor and survivor severe septic patients on demographic and clinical characteristics at the time of severe sepsis diagnosis.

Characteristic	Non–Survivors (*n* = 104)	Survivors (*n* = 206)	*p*–Value
Sex female—*n* (%)	36 (34.6)	66 (32.0)	0.70
Ischemic heart disease—*n* (%)	10 (9.6)	21 (10.2)	0.99
Diabetes mellitus—*n* (%)	42 (40.4)	52 (25.2)	0.009
COPD—*n* (%)	13 (12.5)	28 (13.6)	0.86
Chronic renal failure—n (%)	11 (10.6)	12 (5.8)	0.17
SOFA score—median (p 25–75)	11 (9–14)	9 (7–11)	<0.001
Platelets (cells/mm^3^)—median × 10^3^ (p 25–75)	133 (68–225)	197 (131–271)	<0.001
PaO_2_/FIO_2_ ratio—median (p 25–75)	169 (103–240)	180 (123–270)	0.17
Leukocytes (cells/mm^3^)—median × 10^3^ (p 25–75)	14.9 (6.8–20.4)	14.4 (9.1–18.9)	0.84
Lactic acid (mmol/L)—median (p 25–75)	3.56 (1.60–6.00)	2.00 (1.15–3.50)	<0.001
INR—median (p 25–75)	1.42 (1.15–1.90)	1.25 (1.10–1.50)	0.003
Creatinine (mg/dL)—median (p 25–75)	1.63 (1.00–2.95)	1.30 (0.80–2.10)	0.007
Bilirubin (mg/dL)—median (p 25–75)	0.94 (0.50–2.17)	0.87 (0.47–1.40)	0.26
aPTT (seconds)—median (p 25–75)	36 (29–46)	32 (28–39)	0.005
APACHE–II score—median (p 25–75)	23 (19–28)	19 (15–23)	<0.001
Age—median years (p 25–75)	64 (56–74)	60 (47–69)	0.003
Bloodstream infection—*n* (%)	17 (16.3)	30 (14.6)	0.74
Empiric antimicrobial treatment adequate			0.72
Unknown due to negative cultures—*n* (%)	55 (52.9)	105 (51.0)	
Adequate—*n* (%)	42 (40.4)	83 (40.3)	
Inadequate—*n* (%)	3 (2.9)	4 (1.9)	
Unknown due to antigenuria diagnosis—*n* (%)	4 (3.8)	14 (6.8)	
Microorganism responsibles			
Unknwon—*n* (%)	55 (52.9)	105 (51.0)	0.81
Gram-positive—*n* (%)	26 (25.0)	50 (24.3)	0.89
Gram-negative—*n* (%)	23 (22.1)	51 (24.8)	0.67
Fungii—*n* (%)	4 (3.8)	4 (1.9)	0.45
Anaerobe—*n* (%)	1 (1.0)	2 (1.0)	0.99
Site of infection			0.75
Respiratory—*n* (%)	61 (58.7)	118 (57.3)	
Abdominal—*n* (%)	26 (25.0)	58 (28.2)	
Neurological	1 (1.0)	4 (1.9)	
Urinary—*n* (%)	5 (4.8)	11 (5.3)	
Skin—*n* (%)	5 (4.8)	9 (4.4)	
Endocarditis—*n* (%)	5 (4.8)	6 (2.9)	
Osteomyelitis	1 (0.9)	0	
Substance P (pg/mL)—median (p 25–75)	278 (113–429)	422 (314–522)	<0.001
TNF-α (pg/mL)—median (p 25–75)	36 (18–74)	30 (19–49)	0.24
Interleukin-10 median pg/mL (p 25–75)	38 (8–118)	10 (5–37)	<0.001

COPD = Chronic Obstructive Pulmonary Disease; SOFA = Sepsis-related Organ Failure Assessment; PaO_2_/FIO_2_ = pressure of arterial oxygen/fraction inspired oxygen; INR = International normalized ratio; aPTT = Activated partial thromboplastin time; Acute Physiology and Chronic Health Evaluation (APACHE)-II score; TNF = tumor necrosis factor; data are presented as number (percentage) or median (interquartile range).

**Table 2:** Comparisons on evolution of serum levels of substance P, TNF-α and IL-10, and SOFA score during the first week of severe sepsis.

Parameters—Median (p 25−75)	Nonsurvivors	Survivors	*p*
**Day 1**	(*n* = 104)	(*n* = 206)	
Substance P (pg/mL)—median (p 25–75)	278 (113–429)	422 (314–522)	<0.001
SOFA score—median (p 25–75)	11 (9–14)	9 (7–11)	<0.001
TNF-α—median pg/mL (p 25–75)	36 (18–74)	30 (19–49)	0.24
Interleukin-10 median pg/mL (p 25–75)	38 (8–118)	10 (5–37)	<0.001
**Day 4**	(*n* = 65)	(*n* = 206)	
Substance P (pg/mL)—median (p 25–75)	162 (117–199)	292 (201–380)	<0.001
SOFA score—median (p 25–75)	10 (7–15)	6 (3–10)	<0.001
TNF-α—median pg/mL (p 25–75)	35 (26–49)	22 (14–32)	0.001
Interleukin-10 median pg/mL (p 25–75)	13 (6–37)	6 (5–13)	<0.001
**Day 8**	(*n* = 55)	(*n* = 2064)	
Substance P (pg/mL)—median (p 25–75)	124 (107–202)	242 (175–299)	<0.001
SOFA score—median (p 25–75)	10 (6–13)	4 (1–7)	<0.001
TNF-α—median pg/mL (p 25–75)	26 (15–48)	17 (12–29)	0.04
Interleukin-10 median pg/mL (p 25–75)	12 (6–28)	5 (5–9)	0.001

p 25−75 = percentiles 25–75; SOFA = Sepsis-related Organ Failure Assessment score; TNF = tumor necrosis factor.

**Table 3:** Correlations between serum levels of substance P, TNF-α and IL-10, and SOFA score during the first week of severe sepsis.

Header	Day 1	Day 4	Day 8
SOFA score	*R* = −0.09; *p* = 0.12	*R* = −0.18; *p* = 0.003	*R* = −0.12; *p* = 0.06
TNF-α (pg/mL)	*R* = 0.11; *p* = 0.13	*R* = 0.05; *p* = 0.59	*R* = 0.08; *p* = 0.42
Interleukin-10 (pg/mL)	*R* = 0.10; *p* = 0.19	*R* = 0.03; *p* = 0.72	*R* = 0.13; *p* = 0.18

TNF = tumor necrosis factor; SOFA = Sepsis-related Organ Failure Assessment score. After Bonferroni correction only *p* < 0.005 are statistically signifcant.

**Table 4:** Receiver operation characteristic analysis using serum Substance P levels at days 1, 4, and 8 as predictor of mortality at 30 days in severe septic patients: sensitivity, specificity, positive and negative likelihood ratios, positive and negative predicted values.

Header	Day 1	Day 4	Day 8
Cut-off of Substance P (pg/mL)	<339	<203	<148
AUC, 95% CI, and *p*-value	0.71 (0.65–0.76) *p* < 0.001	0.78 (0.73–0.83) *p* < 0.001	0.74 (0.68–0.79) *p* < 0.001
Sensitivity and 95% CI	66.4 (56.4–75.3)	81.5 (70.0–90.1)	67.3 (53.3–79.3)
Specificity and 95% CI	69.4 (62.6–75.6)	74.3 (67.7–80.1)	81.1 (75.0–86.2)
Positive likelihood ratio and 95% CI	2.2 (1.7–2.8)	3.2 (2.4–4.1)	3.6 (2.5–5.0)
Negative likelihood ratio and 95% CI	0.5 (0.4–0.6)	0.3 (0.1–0.4)	0.4 (0.3–0.6)
Positive predicted value and 95% CI	52.3 (43.4–61.0)	50.0 (40.1–59.9)	48.7 (37.0–60.4)
Negative predicted value and 95% CI	80.3 (73.7–85.9)	92.7 (87.6–96.2)	90.3 (85.1–94.1)

AUC: area under curve; CI: confidence intervals.

**Table 5:** Multiple logistic regression analyses to predict mortality at 30 days.

Header	Hazard Ratio	95% Confidence Interval	*p*-Value
**Model: Mortality estimated at day 1**			
SP levels < 339 pg/mL at day 1	4.296	2.470–7.471	<0.001
SOFA at day 1	1.165	1.070–1.267	<0.001
Lactic acid (mmol/L) at day 1	1.110	1.008–1.223	0.03
Diabetes Mellitus	1.789	0.989–3.237	0.055
Age (years)	1.022	1.002–1.042	0.03
**Model: Mortality estimated at day 4**			
SP levels < 203 pg/mL at day 4	14.619	6.304–33.900	<0.001
SOFA at day 4	1.118	1.017–1.229	0.02
Lactic acid (mmol/L) at day 4	1.652	1.241–2.198	0.001
Diabetes Mellitus	2.001	0.917–4.365	0.08
Age (years)	1.027	1.001–1.054	0.04
**Model: Mortality estimated at day 8**			
SP levels < 148 pg/mL at day 8	6.003	2.711–13.290	<0.001
SOFA at day 8	1.212	1.111–1.323	<0.001
Lactic acid (mmol/L) at day 8	1.557	0.933–2.599	0.09
Diabetes Mellitus	1.669	0.734–3.793	0.22
Age (years)	1.026	0.997–1.056	0.08

SP = substance P; SOFA = Sepsis-related Organ Failure Assessment.

## Abbreviations

APACHE	Acute physiology and chronic health evaluation
aPTT	Activated partial thromboplastin time
COPD	Chronic obstructive pulmonary disease
FIO2	Fraction inspired oxygen
INR	International normalized ratio
ICU	Intensive care unit
PaO_2_	Pressure of arterial oxygen
TNF	Tumor necrosis factor
SOFA	Sepsis-related organ failure assessment score
SP	Substance P
